# Association between systemic lupus erythematosus and autoimmune thyroid dysfunction in pediatric population: a single center experience

**DOI:** 10.1186/s13052-024-01728-4

**Published:** 2024-09-04

**Authors:** Radwa Ahmed Shamma, Hend Mehawed Soliman, Walaa Abdelfattah, Marwa Ahmed Badawy, Eman Shafik Shafie

**Affiliations:** 1 Pediatrics Department, Faculty of Medicine, Cairo University, Cairo, Egypt; 2 Clinical and Chemical Pathology Department, Faculty of Medicine, Cairo University, Cairo, Egypt

**Keywords:** Thyroid dysfunctions, Juvenile systemic Lupus Erythematosus, Thyroid antibodies, Thyroid autoimmunity

## Abstract

**Background:**

Systemic Lupus Erythematosus (SLE) patients are more likely than the general population to suffer from thyroid illness. The major goal was to assess the thyroid dysfunctions due to immunological factors in Egyptian SLE children and how they are related to the course and severity of the illness.

**Methods:**

Fifty children and adolescents with SLE are included in this cross-sectional observational study. Every patient underwent a thorough physical examination and a comprehensive history taking. An enzyme-linked immunosorbent assay (ELISA) approach was used to evaluate the thyroid profile, anti-thyroglobulin (Anti-TG), and anti-thyroid peroxidase (anti-TPO) antibodies.

**Results:**

Of the 50 patients, the female: male ratio (F: M = 7:1) was 44 females and 6 males (12%). They were between the ages of 5 and 17. Out of the patients, thirty-two (64%) had thyroid dysfunctions, 19 (38%) had euthyroid sick syndrome, ten (20%) had overt hypothyroidism, three (6%) had subclinical hypothyroidism, and none had hyperthyroidism. Of the 50 patients, one (2%) had increased anti-TPO, whereas all other patients had normal anti-TG levels. A statistically significant negative correlation (*p*-value 0.007) was seen between the disease duration and free thyroxine (FT4). Furthermore, a significant negative correlation (*p*-values 0.015 and 0.028) was found when comparing the disease duration with thyroid antibodies (anti-TG and anti-TPO).

**Conclusion:**

In Juvenile Systemic Lupus Erythematosus (JSLE), thyroid dysfunctions can be identified. The disease duration but not its activity was significantly correlated with thyroid antibodies. For children with JSLE, thyroid function testing should be done on a regular basis. It is preferable to carry out additional thyroid antibody tests when necessary.

**Supplementary Information:**

The online version contains supplementary material available at 10.1186/s13052-024-01728-4.

## Background

The chronic autoimmune illness known as systemic lupus erythematosus (SLE) is caused by autoantibodies that target self-antigens, causing tissue and cell destruction and inflammation [1]. The most often impacted organs include the neurological system, joints, blood vessels, heart, skin, lungs, kidneys, and liver [2].

The course of SLE is unpredictable, with flare-ups, or episodes of severe flare-ups, alternating with remissions. Numerous rheumatological conditions, such as SLE, have been linked to thyroid autoimmune illnesses. These conditions are caused by humoral and cell-mediated immune responses that induce tissue damage that is specific to the thyroid [3]. It is possible for the two illnesses to manifest one after the other or concurrently [4].

The first associations between lupus and thyroid disorders were reported in 1961. Subsequent researches have shown that compared to the general population, lupus patients had a higher frequency of thyroid dysfunction [5]. It has been documented that SLE patients are more likely to develop papillary thyroid carcinoma, particularly when thyroid autoimmunity is present [2].

Few research have been done on children, although many have examined thyroid disorders and their etiology in lupus patients in adult [2]. Compared to the general population, individuals with juvenile SLE (JSLE) are more likely to experience thyroid dysfunction. Regarding thyroid disorders in (JSLE) there are no statistics available in Egypt [6].Therefore, we highlight the significance of routine thyroid function testing and monitoring throughout children with SLE follow-ups. Thus, the primary goal of this research is to assess the frequency of thyroid dysfunctions due to immunological factors in Egyptian SLE children by measuring anti-thyroid peroxidase and anti-thyroglobulin antibodies, as well as their relationship to the severity and disease duration.

## Methods

In this cross-sectional observational study, 50 children and adolescents with JSLE were diagnosed by clinical examination and laboratory investigations, ranging in age from 5 to 18, were included. We recruited both males and females. Individuals with overlap syndrome and other rheumatological diseases were not included, nor were those with thyroid abnormalities from other sources or those using drugs that may interfere with thyroid function.

All patients attended to the Specialized Pediatric Hospital’s rheumatology clinic for routine follow-up. Patients meeting the study’s inclusion criteria were taken into consideration after obtaining their parents’ or guardians’ informed consent. Between January and December of 2019, a thorough history and physical examination were conducted, with a focus on the thyroid.

Based on the criteria established by the American College of Rheumatology, patients were diagnosed with SLE. Complete clinical assessments, including anthropometric measurements, tanner staging, and an evaluation of disease activity using the systemic lupus erythematosus disease activity index (SLEDAI) scoring system, were performed on all included patients (Bertsias, et al., 2012).

The following laboratory tests were performed as part of routine follow-up and were taken from the patient files:

- Serum creatinine, BUN, ALT, AST, and CBC and ESR.
- Urine analysis, including the ratio of albumin to creatinine.
- C3, C4 serum complements.
- Double strand DNA (anti-dsDNA) and anti-nuclear antibodies (ANA).

The following laboratory tests were carried out: free triiodothyronine (FT3), free thyroxine (FT4), and serum thyroid stimulating hormone (TSH) for the thyroid profile. While thyroid peroxidase and anti-thyroglobulin antibodies were tested, other autoantibodies (Anti-Smith, Anti-RNP, Anti-Ro and Anti-La) were not because they were not available.

Table 1 shows the normal ranges for TSH, FT3, FT4, anti-TG, and anti-TPO. Table 2 provides an illustration of how thyroid functions are interpreted.

**Table 1:** Normal ranges for thyroid function tests & thyroid antibodies in infants and children

Age	Free T4* (ng/dL)	Free T3 (pg/mL)	TSH (mU/L)	AntiTG (AU/ml)	AntiTPO (AU/ml)
1 to 5 years	0.8 to 1.8	2.73 to 4.95	0.7 to 6.6	Normal < 4 Elevated > 4	Normal < 20 Elevated > 20
6 to 10 years	1.0 to 2.1	2.73 to 4.69	0.8 to 6.0		
11 to 18 years	0.8 to 1.9	2.67 to 4.62	0.6 to 5.8		

T4: thyroxine; T3: triiodothyronine; TSH: thyroid-stimulating hormone; AntiTG: antithyroglobulin antibodies; AntiTPO: antithyroid peroxidase antibodies

**Table 2:** Results of evaluating thyroid function in SLE children

Thyroid functions’ evaluation	SLE *N* = 50 (%)
**Normal**	18 (36%)
**Euthyroid Sick Syndrome** (Low FT4, normal, or low FT3, normal TSH)	19 (38%)
**Subclinical hypothyroidism** (Normal FT4 and FT3 with high TSH)	3 (6%)
**Overt hypothyroidism** (Low FT4, normal or low FT3, and high TSH)	10 (20%)
**Hyperthyroidism** (High FT3, FT4)	0

### Assay

Thyroid-peroxidase and anti-thyroglobulin antibodies were measured using the enzyme-linked immunosorbent assay (ELISA) method. An indirect solid phase enzyme immunometric assay (ELISA) method (CTK Biotech, inc.ca 92121, USA) was used to determine FT4 and FT3.

Thyrotropin and TSH concentrations in human serum or plasma was measured quantitatively using the immunoenzymatic colorimetric technique known as TSH ELISA (DiaMetra S.r.l. Headquater: Via Garibaldi, 18-20090 SEGRATE (MI) Italy).

Using the enzyme-linked immunosorbent test (ELISA), we measured the anti-thyroglobulin (anti-TG) and thyroid peroxidase autoantibodies (anti-TPO) (DiaMetra S.r.l. Headquater: Via Garibaldi, 18-20090 SEGRATE (MI) Italy).

### Statistical analysis

The statistical software for the social sciences (SPSS) version 25 (IBM Corp., Armonk, NY, USA) was used to code and enter the data. For quantitative data, the mean, standard deviation, median, minimum, and maximum were used to summarize the data; for categorical data, the frequency (count) and relative frequency (%) were used. The non-parametric Mann-Whitney test was utilized to do comparisons between quantitative variables [7].Chi-square test was done to compare categorical data. When there was an expected frequency of fewer than five, the exact test was conducted [8]. Using the Spearman correlation coefficient [9], correlations between quantitative variables were conducted. Statistical significance was defined as *P*-values less than 0.05.

## Results

50 patients with juvenile systemic lupus erythematosus were included in this cross-sectional study. With a female to male ratio of 7:1, there were 6 male patients (12%) and 44 female patients (88%). Table 3 shows that the patients’ ages varied from 5 to 17 years, with a median age of 12 years, and their diagnostic ages ranged from 2 to 13.5 years, with a median age of 9.75 years.

**Table 3:** Demographic and clinical characteristics of study group (*N* = 50)

	Median	Minimum	Maximum
**Age of patients in years**	12	5	17
**Age of diagnosis in years**	9.75	2	13.5
**Duration of disease in month**	18	3	163
**Height (cm)**	140	100	167
**Height standard deviation**	-1.5	-6.6	1.34
**Weight (kg)**	36	10	75
**Weight standard deviation**	− 0.56	-4.70	2.1
**Body Mass Index (BMI) kg/m2**	19.5	15	38
**BMI standard deviation**	0.46	-2.8	2.29
**Systolic blood pressure (mmhg)**	100	80	135
**Diastolic blood pressure (mmhg)**	70	55	100

Malar rash was the most common symptom of SLE at diagnosis (68%) of patients. Arthritis followed (66%) of cases, photosensitivity (62%), and nephritis (52%) of cases. Moreover, no patients displayed discoid rash.

Just two individuals (4%) showed severe disease activity based on the systemic lupus disease activity index (SLEDAI), whereas 9 (18%) patients had mild to moderate disease activity. Twenty-six (52%) of the study group’s participants had low FT3, 25 (50%) had low FT4, and 19 (38%) had excessive TSH in relation to thyroid parameters. Furthermore, among the 50 patients in the study group, 1 (2%) showed elevated anti-TPO levels, whereas the remaining patients had normal anti-TG levels.

As shown in Tables 2 and 32 patients (64%) had thyroid dysfunctions, 19 patients (38%) had euthyroid sick syndrome, 10 patients (20%) had overt hypothyroidism, 3 patients (6%) had subclinical hypothyroidism, and 0 patients had hyperthyroidism.

The most common symptoms of hypothyroidism that were reported were fatigue in 42% of patients and depressed mood in 38%, followed by dry skin in 18%, and no patients developed goiter.

There was not a statistically significant association between disease activity (as measured by the SLEDAI score) and either the patients’ age or the disease duration, with *p*-values (0.929) and (0.831), respectively.

When we compared the SLEDAI disease activity score and the patients’ treatment, we discovered that there is a significant (*p*-value 0.034) correlation between the disease activity and cyclophosphamide use. When we compared thyroid function (FT3, FT4 and TSH) and thyroid antibodies (anti-TG, anti-TPO) with disease activity (using the SLEDAI score), we did not find any statistically significant correlation.

By comparing patients with thyroid disorders and SLEDAI disease activity scoring we found 32 patients with abnormal thyroid functions, 25 patients (78.1%) had no activity, 5 patients (15.6%) had mild to moderate activity and 2 patients (6.3%) had severe activity. There was no significant relation between disease activity and thyroid disorders with *P* value of 0.062.

**Fig. 1:**
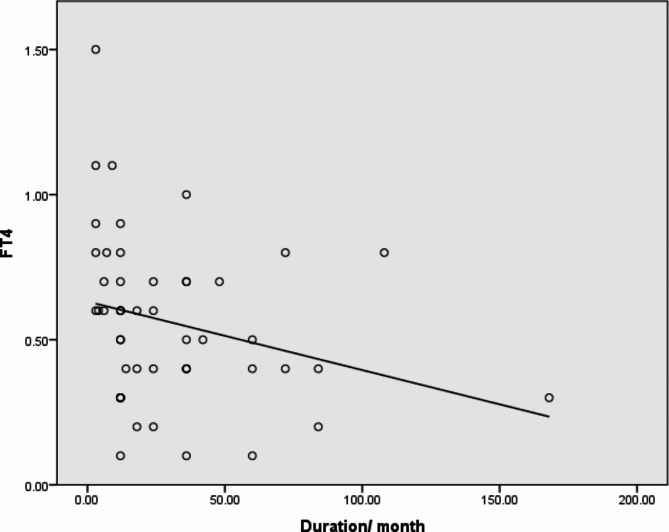
Significant negative correlation between FT4 and duration of the disease. *R*= -0.377,** P 0.007**

**Fig. 2:**
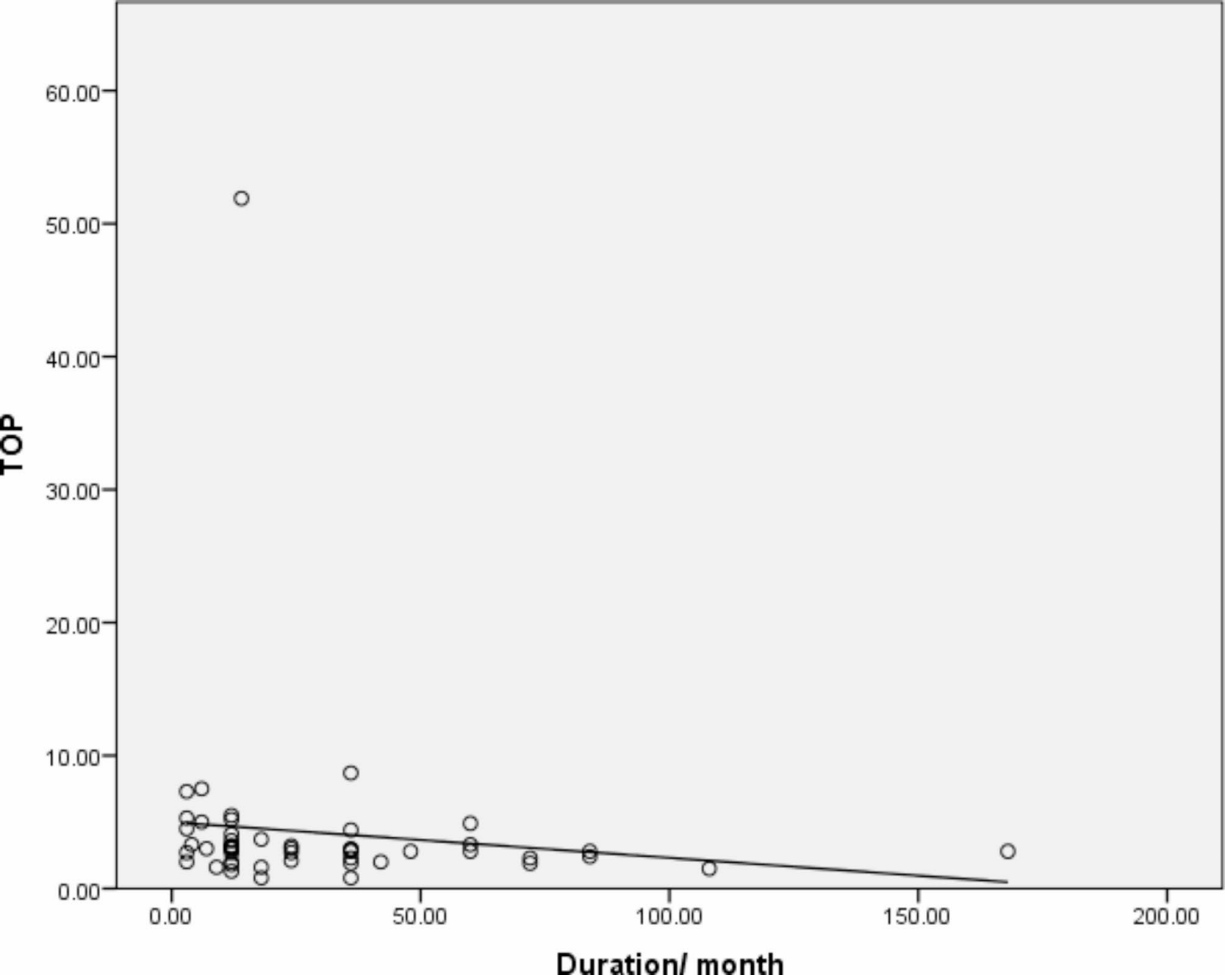
Significant negative correlation between anti-TPO and duration of the disease. *R*= -0.311,*P* = 0.028

When we compared the disease duration with thyroid functions (FT3, FT4), we found a strong negative correlation (*p*-value 0.007) between FT4 and disease duration, as illustrated in Fig. 1. Additionally, as Fig. 2 illustrates, we discovered a significant negative correlation between the disease duration and thyroid antibodies (anti-TG, anti-TPO), with *p*-values 0.015 and 0.028, respectively.

When we compared thyroid disorders with SLE manifestations, no significant correlations was found between them. And also by comparing, the age of patient and disease duration with thyroid disorders, no significant correlation between each of them and thyroid disorders was found (p-values (0.09) and (0.05), respectively).

There was no significant correlation between thyroid functions, thyroid antibodies and laboratory investigations including complete blood count and renal functions.

By comparing thyroid disorders with treatments which were used among studied group we found that out of 32 patients with thyroid disorders 25 patients (78%) were using cyclosporine with significant relation between using cyclosporine and thyroid disorders with *p* value of 0.041.

## Discussion

Among the 50 patients in this study, 1 (2%) showed elevated anti-TPO levels, whereas the remaining patients had normal anti-TG levels. This contradicts numerous other studies that discovered elevated levels of anti-TG, including Abd-Elnabi et al. [10], which showed anti-TG in 8 patients (20%). Additionally, even in SLE patients without clinical thyroid dysfunction, anti-TG has been detected more frequently in SLE patients than in the general population [11]. Furthermore, this is consistent with the research conducted by Assal et al. [12], who examined 30 JSLE patients and discovered 6% of them had anti-TG.

In our study, 32 patients (64%) had thyroid dysfunctions, 19 patients (38%) had euthyroid sick syndrome, 10 patients (20%) had overt hypothyroidism, 3 patients (6%) had subclinical hypothyroidism, and 0 patients had hyperthyroidism. While **Abdel-Nabi study** in 2016 found that there were fourteen cases (35%) with thyroid dysfunctions. Six cases (15%) with euthyroid sick syndrome, four cases (10%) with hypothyroidism, two cases (5%) with hyperthyroidism and two cases (5%) with sub-clinical hyperthyroidism. Another study done on forty children with SLE by **El-Ghoneimy et al.** [3] in 2011, they found that 6 patients (15%) had subclinical hypothyroidism, while the remaining 34 patients (85%) were euthyroid. Other studies had numbers higher than the present study, **Al-Girgawy and Al-Shabrawy** [13] in **2007** found that: 28.90% of JSLE patients showed sub-clinical hypothyroidism; 22.66% showed sub-clinical hyperthyroidism, while 47.37% showed normal results. Also **Madar et al.** [14] in **2006** found thyroid dysfunction and found that the most common abnormality was clinical hypothyroidism (8.3%). Hypothyroidism was detected in 11.6% of SLE patients. None of the patients had evidence of hyperthyroidism.

A further finding from this research is that the SLEDAI mean was comparatively low, indicating that patients with mostly high disease activity would benefit more from closer monitoring of thyroid autoantibody. In SLE patients, anti-thyroid antibodies may exhibit a variable pattern over time. Some individuals during follow-up visits may test negative even though they tested positive at one time in their illness [15].

FT3, FT4, TSH, Anti-TG and Anti-TPO did not significantly correlate with disease activity as determined by the SLEDAI score in this study. This is consistent with a research by **Posselt et al**. [16], which discovered no relationship between autoantibodies or Hashimoto thyroiditis and disease activity or cumulative damage. Also, it is consistent with** Franco et al.** [17], who investigated the incidence and consequences of thyroid autoimmunity and autoimmune thyroid disease (AITD) in SLE patients, finding that autoimmune thyroid disease (AITD) is common in SLE patients and has no relationship to the severity of the disease.

This study revealed non-significant correlation between FT3, TSH and disease duration but a significant negative correlation between patients’ anti-TG, anti-TPO and T4 levels and disease duration. That is similar to **Abd-Elnabi et al.** study [10], which discovered non-significant correlations between FT3, FT4 and disease duration, and a significant negative correlation between patient anti-TG levels and serum TSH and disease duration.

The 50 patients in this study had a female-to-male ratio of 7:1, demonstrating the significance of hormonal variables in the disease’s clinical manifestation. This resembles the study on the incidence, prevalence, and sex distribution of JSLE conducted by **El-Gamasy and El-Naghy** [18]. Girls had a greater prevalence. Out of the 80 SLE patients, 70 (87.50%) were female and 10 (male), with F: M ratio of 7:1. It is also comparable to **El-Ghoneimy et al.‘s (2011)** study on the JSLE pattern in Egypt. A female to male ratio of 7:1 was seen among the 34 patients under research; 4 patients (12%) were male, and 30 patients (88%) were female. Differences in ethnic variables and genetic backgrounds could be the cause of this, or it could be related to the varied numbers of patients under the study.

In this study, Malar rash was the most common symptom of SLE at diagnosis (68%) of patients. Arthritis followed (66%) of cases, photosensitivity (62%), and nephritis (52%) of cases. Moreover, no patients displayed discoid rash.

This is consistent with **Jebali et al.** research [19] who found that the most prevalent presentation was mucocutaneous (67% of patients) and musculoskeletal (86% of patients), with neurological disorders being the least common symptom. Furthermore, it has similarities to the research on thyroid dysfunction and autoantibodies in Egyptian SLE patients. The most common symptoms of SLE that they found were serositis in (30% of patients), fever in (70% of patients), photosensitivity in (80% of patients), arthritis in (50% of patients), and malar flush in (90% of patients) [20].

Therefore, as a component of the SLE patient’s clinical profile, especially if they have thyroid antibodies, we advise the periodic screening of thyroid antibodies and functions and to detect clinical/subclinical thyroid illness [21]. When diagnosing hypothyroidism, screening for connective tissue disease should also be considered.

## Conclusion

In Juvenile Systemic Lupus Erythematosus, thyroid dysfunctions can be identified. The disease duration but not its activity was significantly correlated with thyroid antibodies. For children with JSLE, thyroid function testing should be done on a regular basis. It is preferable to carry out additional thyroid antibody tests when necessary.

### Limitations

There was a small sample size in this investigation. To further assess the prevalence of thyroid dysfunction in patients with SLE, more long-term prospective cohort studies are required.

## Electronic supplementary material

Below is the link to the electronic supplementary material.

Supplementary Material 1

## Data Availability

The corresponding author can provide the datasets created and/or analyzed during the current work upon reasonable request.

## Abbreviations

AITD: Autoimmune Thyroid Disease
ALT: Alanine Transaminase
ANA: Anti-Nuclear Antibodies
Anti-dsDNA: Anti-double strand DNA
Anti-TG: Anti-Thyroglobulin Antibodies
Anti-TPO: Anti-Thyroid-Peroxidase Antibodies
AST: Aspartate Transaminase
BUN: Blood Urea Nitrogen
CBC: Complete Blood Picture
ELISA: Enzyme-Linked Immunosorbent Assay
ESR: Erythrocyte Sedimentation Rate
FT3: Free Triiodothyronine
FT4: Free Thyroxine
JSLE: Juvenile Systemic Lupus
TSH: Thyroid Stimulating Hormone
SLE: Systemic Lupus Erythematosus
SLEDAI: Systemic Lupus Disease Activity index
SPSS: Statistical Package for the Social Sciences
