# Supplementary material for: Association between systemic lupus erythematosus and autoimmune thyroid dysfunction in pediatric population: a single center experience
Source: Ital J Pediatr. 2024 Sep 4;50:163. doi: 10.1186/s13052-024-01728-4 (PMC11373283; doi:10.1186/s13052-024-01728-4)
Supplement: Supplementary file 1 — Supplementary Material 1 [file 13052_2024_1728_MOESM1_ESM.docx]

Dear Dr. Claudio Maffeis

Thank you for your letter below, and the reviewers’ comments. We reply to each of these point by point and enclose an amended manuscript which we hope you will now find acceptable for publication.

Thank you for considering our work for “Italian journal of pediatrics”

Regards,

Dr. Eman Shafie

ITJP-D-24-00100
Association between Systemic Lupus Erythematosus and Autoimmune Thyroid Dysfunction in Pediatric Population: A single center experience

Reviewer reports:

Reviewer #1:

Are there any significant difference between patients with or without thyroid disease and positive autoantibodies?

*Authors’ reply: Thank you for your valuable comment* .*There was no significant difference between patients with or without thyroid disease and positive autoantibodies*

Did you find any correlation between thyroid involvement and tTG and AGA deamidated and/or ICA, anti-GAD, anti-Zn-T8?

*Authors’ reply: Thank you for your valuable comment*. *There was no significant correlation between thyroid involvement and thyroid antibodies*

Reviewer #2:

In the methods section I would add the section “assay” and I would remove the section “estimation of TSH”. As a result of this change, the assay sentences (lines 93 and 96) will therefore be part of the specific section “assay”, whereas line 103 will be placed in the previous section.

*Authors’ reply: Thank you for your valuable comment. We are very sorry for the inconvenience. The sentence has been corrected.*

is there a correlation between the use of cyclophosphamide and the thyroid results?

*Authors’ reply: Thank you for your valuable comment. No correlation was found between the use of cyclophosphamide and the thyroid results but there was significant relation between the use of cyclosporine and the thyroid disorders (a paragraph is added for this finding in the result section).*

-	Bring forward lines 142-145 after line 137 and improve the presentation of this result

*Authors’ reply: Thank you for your valuable comment. We are very sorry for the inconvenience. The sentence has been corrected.*

-	Rephrase lines 146-150 and present them in a different way.

*Authors’ reply: Thank you for your valuable comment. The sentence has been rephrased.*-

In general, the discussion is not incisive: it is not very linear and often repeats data already expressed in the results section (as for line 157, lines 167-170, lines 173-174). The first sentence should summarize the results of the work; starting from this the section should evolve into an organic discussion of the results compared with previous studies.

*Authors’ reply: Thank you for your valuable comment. Discussion has been revised and changed*

-	In lines 180-183 the authors try to explain why their patients did not have anti-thyroid Abs as reported in other studies, and blame the use of glucocorticoids. Such an interpretation brings up further questions: for instance, were the patients in the other cohorts not on glucocorticoid therapy? This topic needs to discussed in more depth.

*Authors’ reply: Thank you for your valuable comment. It has been revised and corrected*

-	Rephase lines 184-187

*Authors’ reply: Thank you for your valuable comment. The sentence has been rephrased.*-

-	From line 188 to line 207 the authors present a list of studies, some in agreement and others in disagreement with their results, without any logical connection. It instead seems necessary to focus on the comparison between the results of their study and those of the known literature.

*Authors’ reply: Thank you for your valuable comment. It has been revised and corrected*

-	In the conclusion the authors declare that it is cost-effective to perform biochemical screening of thyroid function as routine for children with SLE. It is however unclear which kind of economic study has been carried out to sustain this affirmation. If the authors refer to clinical costs, to my knowledge there is still no study that can confirm such a statement. It would be more appropriate to simply “recommend” the screening without engaging in too risky considerations about its financial sustainability.

*Authors’ reply: Thank you for your valuable comment. It has been revised and corrected*

Minor comments:

-	In my opinion, the purpose of the study must be the finally sentence of the background. This does not happen. I suggest place line 70 (a higher prevalence…) after reference 5 in line 66. Similarly, I suggest to place line 71 (Thus, we emphasize…) after reference 2, line 68.

*Authors’ reply: Thank you for your valuable comment. It has been revised and corrected*

-	Line 83: a reference is necessary after criteria

*Authors’ reply: Thank you for your valuable comment. Reference of criteria has been added*

-	Line 88: please explain the acronym SLEDAI

*Authors’ reply: Thank you for your valuable comment. It has been added*

-	Rephrase lines 123-124

*Authors’ reply: Thank you for your valuable comment. The sentence has been rephrased.*-

-	Remove the brackets (and their contents) from lines 125-128 as they are all already specified in table 2

*Authors’ reply: Thank you for your valuable comment. It has been revised and corrected*

-	In line 133 the p values should be in brackets

*Authors’ reply: Thank you for your valuable comment. Brackets has been added*

-	Lines 156 use JSLE and not Juvenile SLE (as explained in line 154)

*Authors’ reply: Thank you for your valuable comment. It has been revised and corrected*

-	Combine tables 2 and 4

*Authors’ reply: Thank you for your valuable comment. It has been revised and corrected*

-	Table 3: duration about what?

*Authors’ reply: Thank you for your valuable comment. Duration of disease has been added*

blood pressure is lacking (mmHg) after diastolic

*Authors’ reply: Thank you for your valuable comment. (mmHg) after diastolic has been added*
